# Clerical Marriages

**Published:** 1876-04

**Authors:** 


### CLERICAL MARRIAGES.

Some irate individual has worked himself up to fever-heat, and the next very natural step of fault-finding, in the daily papers, that young clergymen would marry almost as soon as they obtained their "license," with the doleful result, in a great many cases, of a houseful of children on a starvation salary. Perhaps this gentleman was so ugly or so shamefaced, that he could get no body worth having to marry him; for of all persons in the world, girls of any kind of spirit have a contempt for "sheepish" young men and small-soul calculating people. Maybe he was more successful in getting a wife than children; and took to the tactics of the Fox without a rudder. Be that as it may, every liberal-minded man will cordially admit, that as the clergy, as a class, are the best educated, the most influential, as well as the most unexceptionable in their deportment in the relations of life, whether as citizens, neighbors or kindred, they are richly entitled to all the privileges and immunities of law-abiding men, whether they be candidates for matrimony or for Congress. Let young clergymen take their chances, and if they have to pay a good price for the same, if the whistle costs them dear, it is their own look-out. "As they mak their bed, so they maun lee doon."

It is particularly doleful to have a whole houseful of children, and not have the wherewithal to support them in any kind of respectability; but then there is something in the doctrine of compensations; and who shall deny that a dear, delightful, sweet-tempered wife, as neat as a new pin, as prudent as Miss Prim; as firm as a rock in the right; as pure as the dew-drop, and as smiling as the morning, is worth more than a houseful of children, and will counterbalance many of the inconveniences of too much property of that sort, and not enough of another kind?

In important respects, a man without a wife is of "no account." He has very little "position," either in society or in the financial world, because it is felt that there is nothing to fix him; nothing to prevent him any day from spiriting himself away to "parts unknown." These items hold good as to clergymen, while their relations are such, that being unmarried, they are disqualified from the unexceptionable performance of some of their duties; making them liable to be thrown into many embarrassing situations; besides, they can not truly and properly sympathize with those who are married and have children, and are sore vexed, and "careful about many things."

But any thing can be done which, under the circumstances, ought to be done, and the following straight shoot between Scylla and Charybdis is proposed:

Whereas, a man without a woman is "a poor shoat" any how; and

Whereas, he is a baby, without a wife, and is always in a fret or a stew or most annoying unfixedness, being in "a strait betwixt two," (a wife or no wife); and

Whereas, a state of betweenity is as uncomfortable as a locality upon the points of two horns of a dilemma; and

Whereas young clergymen will get married, whether it is prudent or advisable or not;

Let it be permitted that they marry immediately if not sooner, with the following sole restriction:

That no unmarried clergyman be allowed to take to wife any one who is not a maiden of a third of a century in age.

The advantages of such an arrangement are neither few nor small, while the disadvantages are not important.

There would most probably be no more than three or four children.

The chances would be very great that the mother would have the health, strength and vigor to perform to them all of a mother's duties, live to see them settled in life, and still not be very old.

The minister would then have a wife whose maturity of judgment, whose force of character, and whose bodily energy would enable her to be to him a counselor, a sustainer, a housekeeper—thereby not only being a help-meet for him, in his official capacity, but by relieving him of all household cares, he would have leisure to devote himself entirely to his more appropriate duties.

A woman at the age of thirty-three and a third years, who has never been married, is considered *passée;* is called an "old maid," and the term is most unjustly used in derision. The very fact of being an old maid is *prima facie* evidence of the possession of purity, prudence and self-denial, and these are essential to the character of a perfect wife; without them, no woman is worth having.

Being an "old maid," implies decision of character; neither shams nor shows nor courtly manners nor splendid persons have won them over; nor fair promises nor shallow tears; they looked beyond the manner and the dress, and finding no cheering indication of depth of mind and sterling principles, they gave up the specious present for the chance of a more solid future, and determined in hope and patience and resignation, to "bide their time."

It is this firmness of purpose, this trait of the mind which looks at future utility, more steadily than at present gratification, that makes all the difference between a mother who is priceless, or of nothing worth. She can not gratify her child or herself to-day, for the reasonable chances of an ill to-morrow. The too-yielding mother has been the utter ruin of multitudes, who else might have been an honor to their kith and kin and country.

In a hygienic point of view, the advantages of marrying a woman who is mature in body, in age, in mind, in judgment, in culture, can not be easily computed; they are beyond measure. On the other hand, for a mere girl to become a mother, is to give up almost every chance of health, and peril life itself; is to throw away a decade of joyous youth, of delicious anticipations "long drawn out" in gladness, growing the sweeter in their expectancy, making of girlhood a lengthened sabbath of sunshine, which ending in a wise marriage, is looked back upon to the close of life with the most delightful associations, yet not regretfully, a deeper sweetness being in the present—all this is thrown remorselessly away, by her who marries too early.

The subject may not be better closed, than by recording the sentiments of a noted name in behalf of that class, from among whom this article recommends the clergy to wed. "I have no sympathy with that rude, unfeeling, and indelicate phrase, *old maid*, which is bandied about in the mouths of rude, unfeeling, and indelicate persons. It is true, that a selfish nature, cut off from all duties and ties, and sinking back into the solitary life of a selfish heart, becomes most unlovely and useless. But shall the few cloud the true nobleness of the many? How many elder sisters, it may be unblessed with outward comeliness, have entered into a brother's or a sister's family, and accepted all its cares as the duty of their life, and, joining hands with the mother, given to each child, as it were, two souls of love, like two wings of God, to help it fly up withal from weakness and ignorance to manhood and strength! How many have cheerfully given up their own whole life, built no nest, sought no companion, but sang in the tree, and near the younglings of another's nest, patient in toil, watchful and laborious in sickness, frugal amidst poverty, rich in nothing but good works, and in these abounding in wealth! When the roll is read above, and they are named that lived in self-sacrifice, in gentleness, in patience, in love, and in the only triumph of disinterested mercy, they who are unmarried and childless, that they might more heroically serve the households of others, and become mothers of children not their own, shall stand high and bright."

